# Design and Performance Comparison of Polymer-Derived Ceramic Ambigels and Aerogels

**DOI:** 10.1021/acsomega.3c04607

**Published:** 2023-08-28

**Authors:** Oyku Icin, Tugce Semerci, Gian Domenico Soraru, Cekdar Vakifahmetoglu

**Affiliations:** † Department of Materials Science and Engineering, İzmir Institute of Technology, 35430 İzmir, Turkey; ‡ Department of Industrial Engineering, University of Trento, Via Sommarive 9, 38123 Trento, Italy

## Abstract

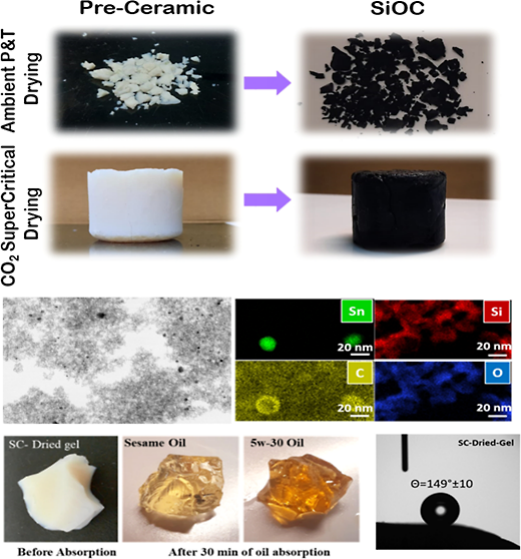

This work reports the synthesis and characterization of preceramic- and polymer-derived SiOC aerogels obtained from a commercial siloxane resin. The preceramic aerogels were obtained by ambient pressure drying (ambigels) and CO_2_ supercritical drying. Despite different drying processes, the final ceramic ambi/aerogels have very similar microstructural features in density, porosity, pore size, and specific surface area. Both materials have shown promising results for oil sorption and water cleaning. Supercritically dried-SiOC aerogel had low thermal conductivity with 0.046 W·m^–1^·K^–1^ at RT and 0.073 W·m^–1^·K^–1^ at 500 °C. These results suggest that substituting the rather complicated and expensive CO_2_–SC drying with the more friendly and cheap ambient pressure drying can be done without having to accept significant microstructural/property degradation.

## 1 Introduction

Aerogels are highly porous materials containing mostly air in their structure (usually above 80 vol %), having submicron pores resulting in medium to high specific surface area (SSA) (∼100–1000 m^2^ g^–1^) and thus relatively low densities below <0.5 g cm^–3^.^1–3^ A number of aerogel types have already been investigated for applications, including catalysis,^4^ sensors,^5,6^ thermal^3,7^ and electric insulation,^8^ wastewater management,^9,10^ energy storage,^11,12^ electromagnetic absorber,^13^ and drug delivery.^14^

In some of the applications mentioned above, exposure to high-temperature and corrosive/oxidative environments during service causes structural failure of the used aerogels.^15^ For instance, one of the most widely investigated silica aerogel shows limited thermal stability above 600 °C (in the air/inert atmosphere), resulting in a pore collapse, particle agglomeration due to shrinkage, and eventual sintering.^16^ Accordingly, the practical utilization of such aerogels in high-temperature applications is minimal. In this regard, polymer-derived (PDC) amorphous silicon oxycarbide (SiOC) aerogels offer advantages due to greater creep resistance, refractoriness, and chemical durability compared to those of silica.^17–19^

A recent comprehensive review of PDC aerogels^20^ reported that SiOC aerogels had been produced predominantly through sol–gel chemistry.^21–25^ Only a few studies focused on directly using commercial preceramic polymers.^1,12,26–28^ Besides, no analysis used the most economical and readily available siloxane resins to obtain aerogels. Compared to the more extensively studied sol–gel process, the commercial preceramic polymer route is economical and faster (no gelation time), delivering high reproducibility. Accordingly, the present study aims to apply such polymers in a facile way to obtain SiOC ambigels and aerogels. The difference relies on the drying procedures; here, the material dried under ambient pressure and temperature is called an ambigel, and the material dried under supercritical conditions is called an aerogel.

## 2 Experimental Procedure

### 2.1 Materials

A commercial poly(methylsilsesquioxane) (PMS) preceramic polymer was obtained from Wacker GmbH (MK Belsil, Wacker GmbH, Burghausen, Germany). Tin(II) 2-ethylhexanoate was purchased from Sigma-Aldrich (tin, Sigma-Aldrich, CAS: 301-10-0, USA) and diluted 15% vol in xylene (ACS reagent ≥99.8%, Merck, CAS: 1330-20-7, Germany). Acetone (extra pure grade, Tekkim, CAS: 67-64-1, Turkey) was used as the solvent. Carbon dioxide (CO_2_, 99.5%) was used for the supercritical drying (SC-dried) process.

### 2.2 Preparation of SiOC Ambi/Aerogels

11.25 g of PMS powder was dissolved in 40 mL of acetone (75 vol % of the prepared blend), followed by tin catalyst addition^29^ (3.75 vol % of PMS precursor). After stirring for an additional 10 min, the solution was transferred into a Teflon liner for the cross-linking conducted in an autoclave (55% filling, at 200 °C for 6 h in a stainless-steel autoclave, 4748 model, Parr, Moline, IL, USA) to prevent solvent evaporation. The autoclave was then cooled to room temperature (RT), and the preceramic wet–gels were transferred into a beaker filled with fresh acetone and washed twice/day for 3 days by renewing the solvent to remove the excess catalyst and unreacted precursors. The preceramic ambigels were obtained by drying at ambient pressure and temperature (AP-dried) and aerogels under CO_2_ supercritical (SC-dried) conditions.

Drying at ambient temperature and pressure took about 2 weeks resulting in ambigel, whereas aerogels were obtained in only 5 h via SC-drying. The wet gels were placed into a high-pressure autoclave (SUPEREX, F-500, 500 mL extractor column, Turkey). When the autoclave temperature reached 50 °C, 30 mL of acetone was added to cover the sample surface and restrict the solvent evaporation from the gel before being in contact with a supercritical CO_2_ condition and to avoid the shrinkage of the solid. Then, CO_2_ was delivered at a certain velocity (5 bar min^–1^) until the pressure of the autoclave reached 150 bar. The SC-drying followed at constant temperature and pressure for about 5 h with the 5 g min^–1^ CO_2_ flow rate, with subsequent degassing performed at a 2–3 bar min^–1^ rate.

All of the dried preceramic ambi/aerogels were finally pyrolyzed at different temperatures (between 600 and 1000 °C) in an alumina tube furnace (PROTERM PTF 16/75/450, Turkey) under Ar flow using the heating rate of 2 °C min^–1^, a flow rate of 200 mL min^–1^, and 2 h dwell time.

### 2.3 Characterization

The morphologies of preceramic and pyrolyzed ambi/aerogels (after sputter-coated with ∼10 nm Au layer) were analyzed by using scanning electron microscopy (SEM; FEI Quanta 250 FEG, Hillsboro, OR, USA) and transmission electron microscopy (200 kV, TEM, Thermo Fisher Talos F200S FEG, Netherlands). The elemental distribution was analyzed by using energy-dispersive spectroscopy (EDS) mapping. Before investigations, the TEM sample was placed on a copper grid covered with amorphous carbon.

Fourier transform infrared spectra (Spectrum Two FT-IR with UATR fitted, PerkinElmer, USA) were recorded in the range of 450–4000 cm^–1^ with 20 scans and 4 cm^–1^ resolution in the transmission mode to investigate structural features of starting precursors, wet and dried preceramic gels, and ceramic ambi/aerogels.

The decomposition behavior of preceramic gels dried at ambient pressure and under CO_2_ supercritical conditions was studied by thermogravimetric analyses (TG/DTA, PerkinElmer Diamond, USA) with a 5 °C min^–1^ heating rate in an N_2_ atmosphere up to 1000 °C.

X-ray diffraction (XRD, Philips X’Pert Pro) data were collected by using the Cu K_α_ radiation (between 2θ; 20–90°, step counting time of 3 s, and scan of 0.05°). The XRD patterns were plotted after normalization.

Nitrogen (N_2_) sorption analyses were performed by a Micrometrics ASAP 2020-Physisorption Analyzer. The samples were degassed at 200 °C for 24 h before the examination. SSA was determined from a Brunauer–Emmett–Teller (BET) approach. The pore size distribution (PSD) and volume were evaluated according to the non-local density functional theory (NLDFT) based on the carbon slit pore model.

The bulk density was measured at RT by Archimedes’ displacement technique using ethanol as a buoyancy fluid. The true densities of samples were obtained using an Anton Paar He pycnometer; accordingly, the total porosities were calculated.^30^

Wetting behavior was analyzed using the theta model contact angle (CA) measurement device (KSV-Attension brand). The water droplet images were taken 30 ms after 5 μL of distilled water droplet was dropped on the samples. The zeta potentials were determined by dynamic light scattering at 25 °C via a Zetasizer Nano ZS (Malvern, UK).

Thermal diffusivity (α) of samples was measured with the laser flash diffusivity method using a Netzsch 467 HyperFlash (Selb, Germany) on square specimens (side length = 10 mm). Measurements were carried out by applying self-regulating pulse widths in the 0.6 to 1 ms range, a laser voltage of 250 V, and a spot amplitude of 3.7 mm within the temperature range from 25 to 500 °C (instrument limit). The penetration model was used to fit the output curve. The total thermal conductivity values (*k*) were calculated using the formula *k* = α × *C*_p_ × ρ, where ρ is the bulk density of aerogel and *C*_p_ is the specific heat capacity. Since the thermal expansion coefficient of amorphous SiOC is very low (∼3 × 10^–6^ K^–1^) up to 1000 °C,^31^ bulk density was assumed constant. The specific heat capacity values (*C*_p_) were extracted from the published data on a similar SiOC system.^31^

Oil absorption experiments were conducted on the polymeric AP-dried and all sets of SC-dried aerogels to eliminate two different oil pollutants from the water. First, the weight of the absorbents was recorded before immersing them in the oil/water emulsion. Then, samples were placed into different oil (sesame and 5W-30 engine)/water emulsions after certain immersion intervals (1 to 90 min) and weighed again. Three independent measurements were conducted for each sample to determine the mean and standard deviation values (±). The absorption capacity was calculated from *Q*_max_ (g/g) = (*m*_f_ – *m*_i_)/*m*_i,_ where *Q*_max_ is the absorption capacity, *m*_f_ is the final weight of sorbent after complete absorption, and *m*_i_ is the initial weight of sorbent. For the regeneration studies, the sample was first placed in a beaker with ethanol for 24 h, followed by oven drying at 100 °C to remove absorbed oil from the sorbent.

## 3 Results and Discussion

### 3.1 Structural

**Figure 1:**
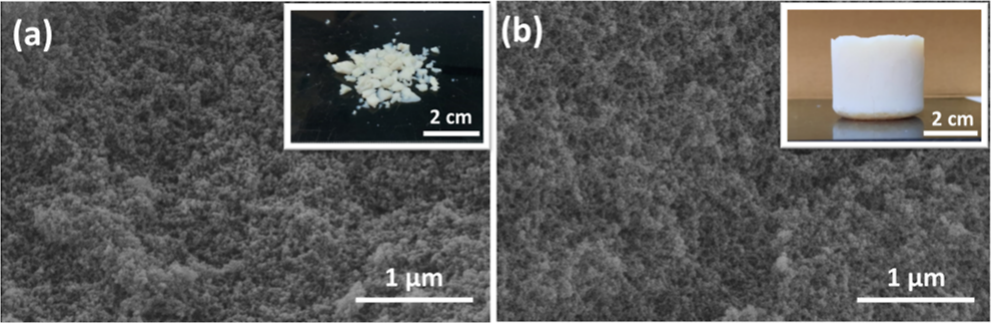
SEM images of the preceramic; (a) ambigel produced via ambient pressure drying and (b) aerogel produced via supercritical drying (the top-right insets show the digital images).

Figure 1 shows the microstructures of preceramic gels dried via AP-drying and SC-drying, as seen in Figure 1 inset, while ambigels were fragmented, monolithic parts (3 cm diameter cylinders) were obtained via SC-drying. Apart from the macro shape, the microstructure of preceramic gels was not significantly altered with the drying technique, a typical aggregation of nanosized or small particles.^12,32^

**Figure 2:**
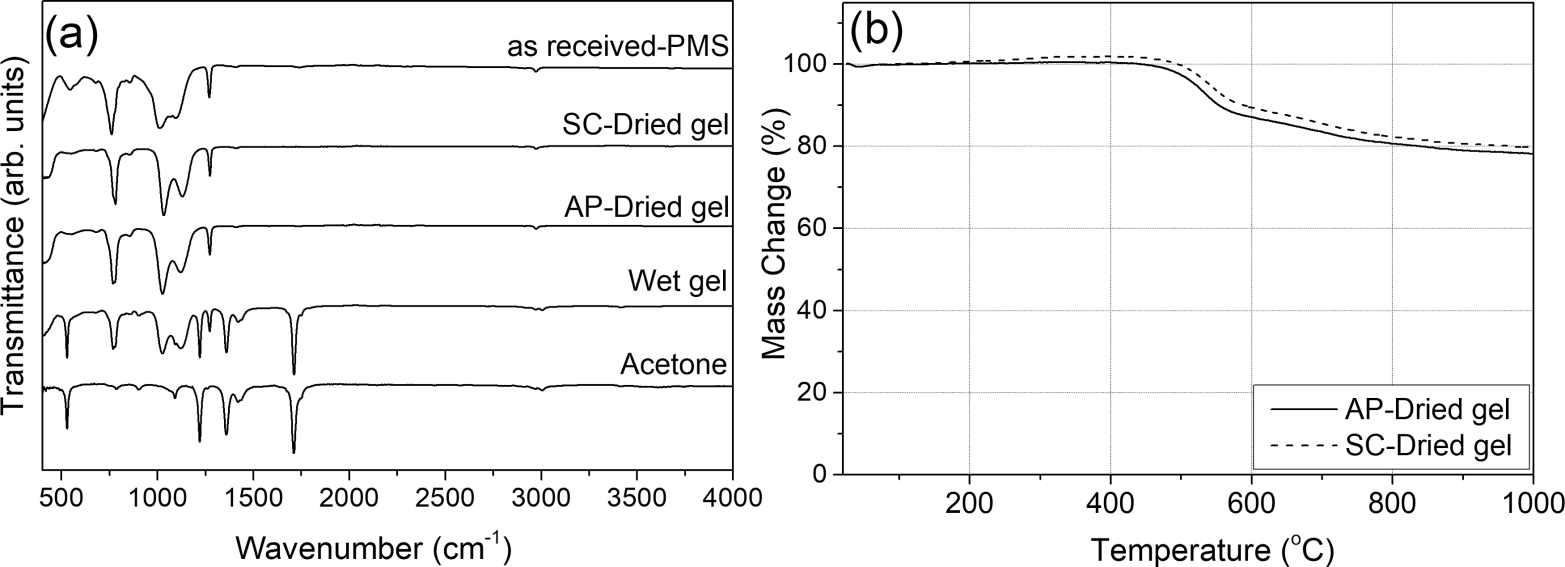
(a) FTIR spectra of precursors, wet and dried preceramic gels, and (b) TGA data of the preceramic ambi/aerogel.

The FTIR spectra of preceramic gels are reported in Figure 2a; for comparison, spectra of acetone, as received PMS, and wet gel (after curing) are also included. In the spectrum of the “as received” PMS polymer, the typical vibration bands of Si–CH_3_ (symmetric) located around 1270 cm^–1^, which is assisted by deformation vibration band at around 760 cm^–1^ (asymmetric Si–CH_3_),^33^ 1100 cm^–1^ (Si–O–Si), 860 cm^–1^ (O–Si–CH_3_), and also the vibration of C–H around 2970 and 550 cm^–1^ are visible.^34,35^ It is clear that after curing, a considerable amount of C=O stretching vibrations 1710 cm^–1^, C–H_2_ vibration 1215, and 1360 cm^–1^ bands related to acetone^36,37^ remained in the case of wet gel. Compared with the wet gel, peaks related to acetone have entirely disappeared for the samples obtained via AP-drying and SC-drying.

TGA results of the dried preceramic gels are given in Figure 2b. The most significant weight loss occurred between 500 and 800 °C because of ceramization. When the temperature was increased to 600 °C, the weight loss of dried preceramic gels was only around 12%. The value increased to ∼18% at 800 °C and finally to 20%, corresponding to a ceramic yield of ∼80% at peak test temperature (1000 °C).

**Figure 3:**
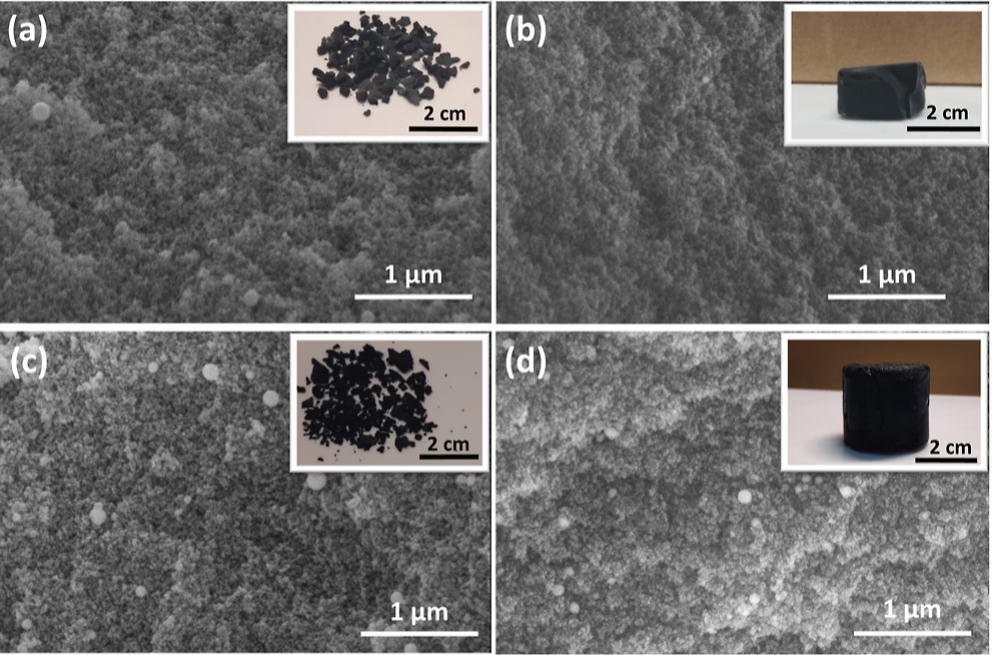
SEM images of (a) ambigel pyrolyzed at 600 °C: AP-dried-600, (b) aerogel pyrolyzed at 600 °C: SC-dried-600, (c) ambigel pyrolyzed at 1000 °C: AP-dried-1000, and (d) aerogel pyrolyzed at 1000 °C: SC-dried-1000 (the top-right insets show the digital images).

SEM micrographs and digital pictures (insets) of the ambi/aerogels after pyrolysis at 600 °C in Figure 3a,b and 1000 °C in Figure 3c,d are given. SC-dried aerogels retained their monolithic structure after pyrolysis but had some degree of shrinkage. Linear shrinkage for SC-dried-600 was ∼6% while it reached ∼24% for SC-dried-1000 upon pyrolysis. In addition, aerogels had different colors depending on the pyrolysis temperature. The CERaMERs (hybrid ceramics or polymer + ceramic structures) were gray-colored when pyrolyzed at 600 °C (Figure 3b; inset),^38^ whereas the amorphous SiOC ceramics were black (Figure 3d; inset) upon 1000 °C pyrolysis as a result of the decomposition of the organic groups and the enhanced free carbon precipitation.^39^

**Figure 4:**
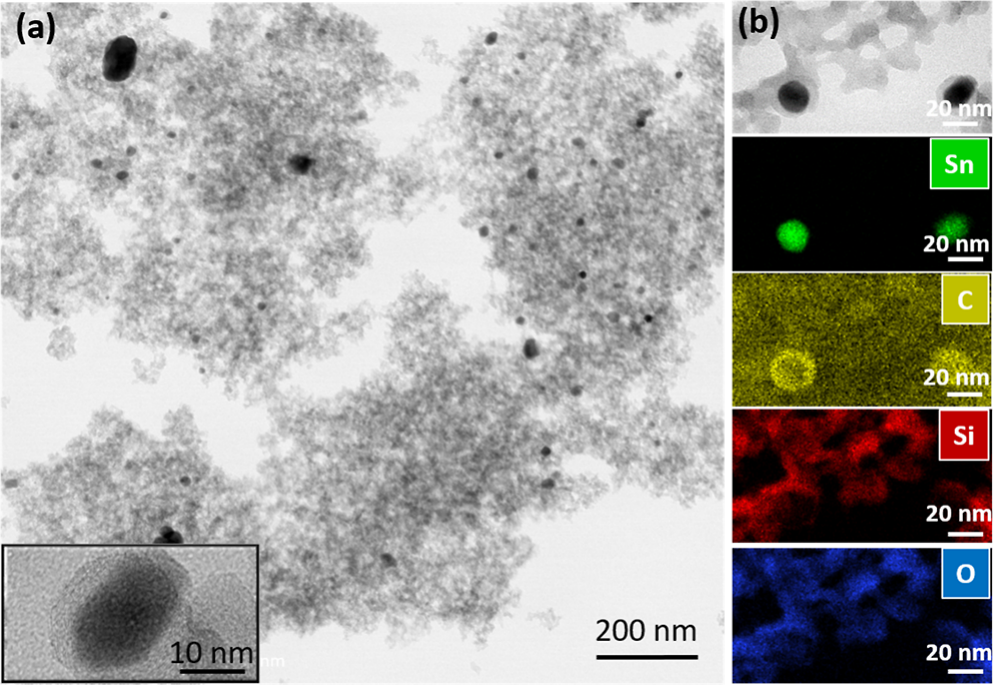
TEM images of (a) aerogel pyrolyzed at 1000 °C: SC-dried-1000 (the left inset shows the Sn nanoparticle dispersed within the amorphous SiOC ceramic matrix) and (b) EDS mapping.

Figure 4 shows the TEM investigations of the SiOC aerogel (SC-dried-1000). The SiOC aerogel structure comprises a highly porous network on which uniformly distributed spherical metallic tin (Sn) nanoparticles (dark regions) are present. This is because liquid tin has poor wettability on SiOC.^40^Figure 4a inset and Figure 4b suggest that a carbon layer encapsulates the precipitated Sn nanoparticles.^41^ According to the EDS mappings (Figure 4b), the amorphous SiOC matrix consisted of only silicon (Si), carbon (C), oxygen (O), and Sn.

**Figure 5:**
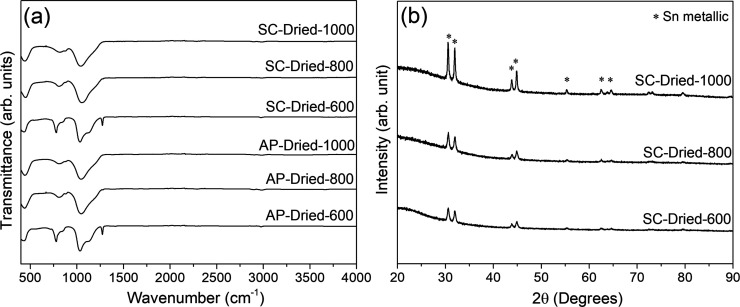
(a) FTIR spectra of both AP-dried and SC-dried samples produced by 600, 800, and 1000 °C pyrolysis, (b) XRD data of the SC-dried samples. At the top of the experimental data, reference reflection marks for metallic Sn (ICDD PDF # 01-086-2264) are given.

The FTIR spectra of the samples pyrolyzed at 600, 800, and 1000 °C are listed in Figure 5a. Typical Si–CH_3_ vibration bands related to the preceramic polymer (at 760 and 1270 cm^–1^) can still be seen for AP-dried-600 and SC-dried-600 samples, disappearing with an increase in pyrolysis temperature. This was evidently due to the incomplete ceramization at 600 °C. All the pyrolyzed samples had a spectrum with the Si–O bond, which belongs to Si–O–Si deformation (at 450 cm^–1^), Si–C and Si–O stretching (at ∼805 cm^–1^), and a broad peak corresponding to Si–O and Si–C stretching due to Si–O–Si/Si–O–C (1035 cm^–1^) vibrations of the silicon oxycarbide network.^33,34,42,43^

The XRD patterns of the SC-dried samples are also shown in Figure 5b. The results showed broad Bragg reflections for 2θ between 10 and 30° related to amorphous SiOC and clear peaks resolved for the metallic tin from the catalyst decomposition and reduction during pyrolysis.^29^

**Figure 6:**
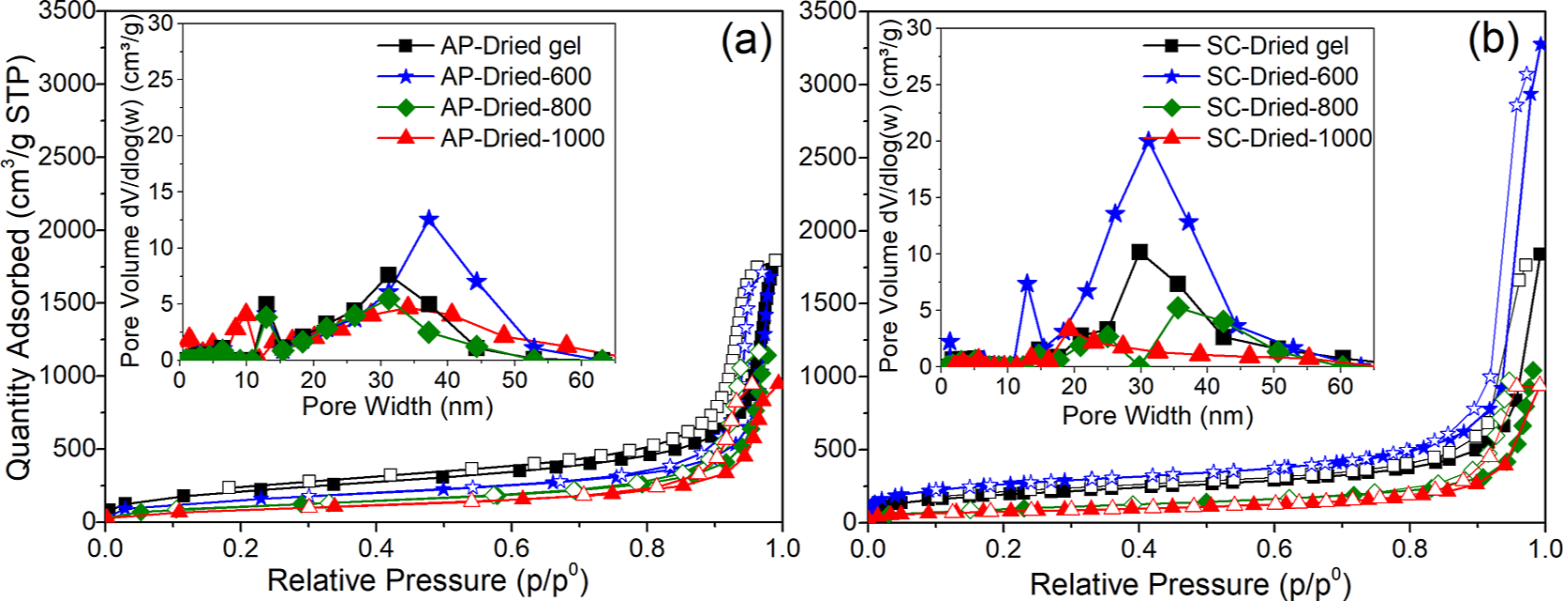
N_2_ sorption isotherms of (a) ambigels (AP-dried series) and (b) aerogels (SC-dried series) (the top-left insets represent the pore size distribution curves).

Figure 6 shows the N_2_ sorption isotherms together with the PSD (data given in the insets) of all ambi/aerogels. The isotherms for the ambigels (Figure 6a) can be classified as type IV according to IUPAC, indicating a hysteresis loop corresponding to mesopore size (also supported with PSD in the inset of Figure 6a). The SSA and pore volume of the samples are also given in Table S1. Among the ambigels, preceramic gel (polymeric AP-dried-gel) showed the highest SSA and pore volume, 783 m^2^ g^–1^ and 2.71 cm^3^ g^–1^, respectively. After pyrolysis at 1000 °C, SSA (318 m^2^ g^–1^) and pore volume (1.45 cm^3^ g^–1^) decreased probably due to the completion of the ceramization followed by the shrinkage (see later the PSD data).

For SC-dried samples, SC-dried-600 had the highest SSA of 917 m^2^ g^–1^ and a pore volume of 4.92 cm^3^ g^–1^. The increased surface area and pore volume could primarily be caused by transient porosity formed from gaseous byproducts during ceramization in such intermediate temperatures.^33^ The PSD data also supported this (see Figure 6b-inset) with peaks around 10–20 nm for SC-dried-600, not present in other samples. It is also important to note that while SC-dried-1000 had a narrower PSD between 15 and 30 nm, AP-dried-1000 had a bimodal PSD with a broader range from 10 to 60 nm and peaks between 1 and 10 nm.

The He pycnometer measurements revealed that the skeletal densities of polymer and ceramic ambi/aerogels ranged between 1.37 and 2.45 g cm^–3^ similar to other studies.^44,45^ The bulk density of polymeric systems (e.g., AP-dried gel with 0.26 g cm^–3^) was lower than that of ceramic ambi/aerogels, e.g., SC-dried-1000 (0.47 g cm^–3^). It is essential to note that while for aerogel systems, the total porosity is generally defined as higher than 80 vol %, for PDC aerogel systems, total porosity values have not been given in previous studies. However, if the pore volumes obtained from N_2_ sorption studies are compared with those for other PDC aerogel systems, it can be seen that the resin-derived ceramic aerogels had higher pore volumes and surface area values^9,12,26,46^ but with total porosities lower than those for the carbon or silica-based aerogels.^47^ Still, the cost of the PMS precursor is around 10 $ per 100 g, i.e., 15 times lower than TEOS regularly used for silica aerogel processing; further investigations are required for appropriate cost and mechanical property analysis.

It is essential to note that while a large extent of the drying stress caused fragmentation for the AP-dried samples, the initial porous solid network remained relatively intact. Besides, it was shown that the pore characteristics of the samples (pore volumes, SSA) were not altered with the drying method followed.

### 3.2 Properties

**Figure 7:**
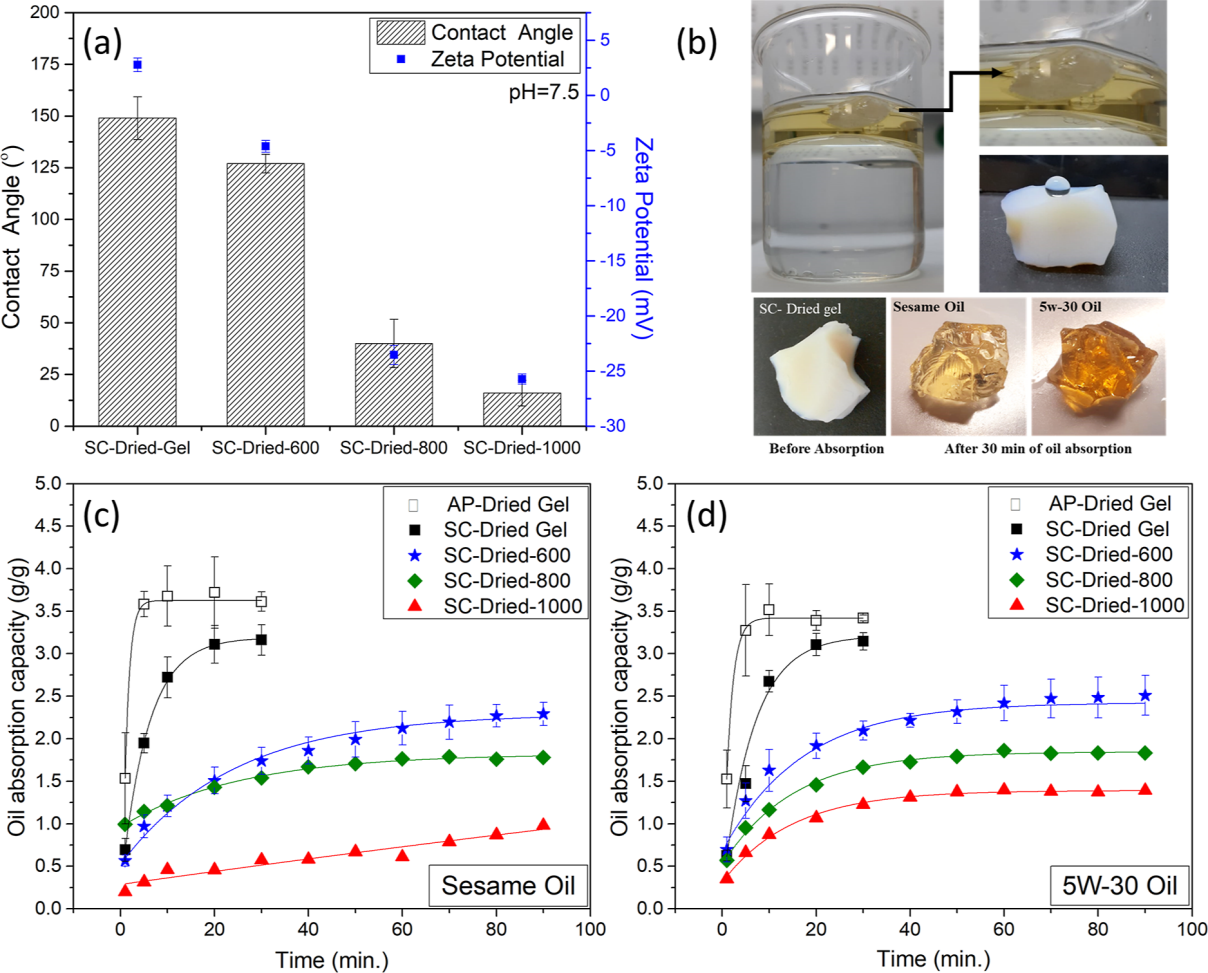
(a) CA and zeta-potential data for SC-dried aerogels, (b) demonstration of oil absorption process, hydrophobicity, and physical appearance of SC-dried gel before and after different oils (sesame and 5W-30) absorption experiment, oil absorption kinetics for (c) sesame oil, and (d) 5W-30 oil (the solid lines represent the nonlinear fitting resulting in *R*^2^ > 0.9788).

Since the formed ambigels were particulates or small-sized chunks (see Figure 1a, inset), the analysis of their surface characteristics was complicated. Accordingly, such tests were applied only on the SC-dried aerogels, as given in Figure 7a, for CA and zeta-potential data.

CA was decreased from 149 ± 10° (polymeric aerogel) to 16 ± 6° (SiOC aerogel) (Figures 7a and S1). Furthermore, the measured CA decreased as the pyrolysis temperature increased, corroborating the disappearance of Si–CH_3_ observed via FTIR. Besides, pyrolysis above 800 °C enhanced the hydrophilicity, due probably to the Si–OH bonds; see as is, the non-normalized FTIR spectrum for SC-dried-1000 in Figure S2. In other words, when the pyrolysis temperature was increased, yielding polymer to ceramic transformation, it caused a change from a hydrophobic surface to a hydrophilic nature.^33,48^

The zeta potentials of the SC-dried aerogels were measured in an ethanol suspension at pH = 7.5. Due to ethyl (nonpolar) groups on the surface and inadequate hydrophilicity, the polymeric SC-dried and SC-dried-600 can only be partially wet by ethanol (polar), forming an unstable suspension and causing low zeta potentials.^49^ Instead, similar to a previous study, upon ceramization negative potential values were recorded as −23.5 ± 0.8 and −25.7 ± 0.4 mV for SC-dried-800 and SC-dried-1000, respectively.^50^

The oil sorption kinetics of the SC-dried aerogel series are shown in Figure 7c,d. The sorption rates are high at the initial 10 min and reach saturation at around 20 min. Among the samples, the polymeric SC-dried gel achieved the highest oil sorption capacity of 3.14 g g^–1^ (sesame oil) and 3.16 g g^–1^ (5W-30 engine oil).

These methyl-based polysiloxane-derived aerogels (see Figure 7b) are inherently hydrophobic, grounding excellent selectivity for oils in water. The oil absorption capacity decreased with the order of 2.51, 1.83, and 1.39 g g^–1^ (sesame oil) and 2.29, 1.78, and 0.98 g g^–1^ (5W-30 engine oil) for 600, 800, and 1000 °C pyrolyzed sample, respectively (Figures 7c,d and S3). The effect of the pyrolysis temperature on the wetting behavior is in line with the previous report.^33^ Meanwhile, a regeneration study was conducted with an SC-dried-600 sample (first cycle capacity of 2.51 g g^–1^ for sesame oil); after second cycle, it had a capacity of 1.49 g g^–1^ (in Figure S3), showing a 28% drop.

**Figure 8:**
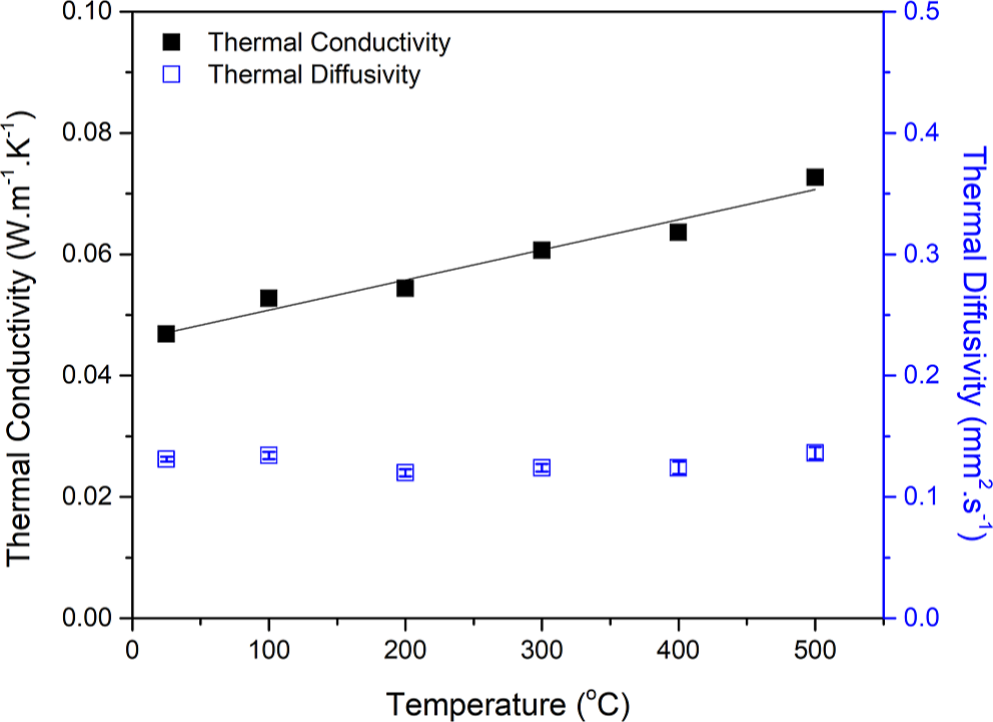
Temperature-dependent thermal conductivity and diffusivity values of the SiOC (SC-dried-1000) aerogel from RT to 500 °C. A bulk density of 0.47 g cm^–3^ was constant at all testing temperatures (the solid line represents the linear fitting resulting in *R*^2^ = 0.9575).

Temperature-dependent thermal diffusivity and conductivity values of the SC-dried-1000 SiOC aerogel are given in Figure 8. The specific heat capacity of SiOC aerogels was subjected to the test temperatures and taken as 0.75–1.12 J·g^–1^·K^–1^ between 25 and 500 °C.^31^ These values were within the expected range for inorganic solids, parallel to the known values for vitreous silica and β-SiC.^31^ Accordingly, the *k* value of 0.046 W·m^–1^·K^–1^ was obtained from a SiOC aerogel with total porosity of 80.5 vol % at RT, which increased with the increase in the measurement temperature.

At a maximum temperature of 500 °C (instrument limit), a *k* of 0.073 W·m^–1^·K^–1^ was noted. This value is 20 times lower than the amorphous dense SiOC presenting a *k* of 1.5 W·m^–1^·K^–1^ at 500 °C.^31^

These conductivity values were lower than those obtained from other preceramic polymer-derived aerogels. For instance, a SiOC aerogel with total porosity of around 80 vol %, *k* of 0.066 W·m^–1^·K^–1^ was measured at RT.^51^ Wang et al.^52^ recently prepared a honeycomb-like SiOC aerogel, possessing high total porosity of 90 vol % and *k* of 0.057 W·m^–1^·K^–1^. Similarly, thermal conductivity between 0.068 and 0.107 W·m^–1^·K^–1^ (at RT) was documented for the sol–gel-derived SiOC aerogels having 76–81 vol % total porosity.^53^ Sol–gel-derived BN/SiOC aerogel composites yield with 0.04–0.2 W·m^–1^·K^–1^ (at RT).^15^

## 4 Conclusions

This study successfully synthesized preceramic ambigels (ambient pressure dried) and aerogels (CO_2_ supercritical dried), which were later pyrolyzed at 600–800–1000 °C to produce SiOC components. While ambigels were fragmented during drying at ambient pressure, monolithic aerogels were obtained through CO_2_ supercritical drying. However, aerogels and ambigels (both polymeric and ceramic forms) yielded a total porosity of around 80 vol %, while the SSA varied between 274 and 783 m^2^ g^–1^ with a pore volume of 1.40–2.70 cm^3^ g^–1^.

Both polymeric and ceramic aerogels were evaluated for the oil sorption potential. The highest capacity of 3.14 g g^–1^ (sesame oil) and 3.16 g g^–1^ (5W-30 engine oil) were achieved for polymeric aerogels due to high porosity (∼80 vol %), surface area, and inherently hydrophobic structure. The temperature-dependent thermal conductivity measurements showed that a supercritically dried aerogel produced at 1000 °C pyrolysis had low thermal conductivity with 0.046 W·m^–1^·K^–1^ at RT and 0.073 W·m^–1^·K^–1^ at 500 °C.

## Supporting Information Available

Properties of synthesized ambi/aerogels, CA images with water droplets on the sample surface, FTIR spectrum with no data normalization, and absorption capacities of ambi/aerogels for sesame and 5W-30 oils (PDF)
