# Supplementary material for: Design and Performance Comparison of Polymer-Derived Ceramic Ambigels and Aerogels
Source: ACS Omega. 2023 Aug 28;8(36):32955–62. doi: 10.1021/acsomega.3c04607 (PMC10500666; doi:10.1021/acsomega.3c04607)
Supplement: Supplementary file 1 — ao3c04607_si_001.pdf [file ao3c04607_si_001.pdf]

Supporting Information for

Design and performance comparison of polymer derived ceramic ambigels and aerogels

Oyku Icin[1], Tugce Semerci[1], Gian Domenico Soraru[2], Cekdar Vakifahmetoglu[1*]

[1] Department of Materials Science and Engineering, İzmir Institute of Technology, 35433, İzmir, Turkey

[2] Department of Industrial Engineering, University of Trento, Via Sommarive 9, 38123, Trento, Italy

Table S1. The properties of synthesized Ambi/Aerogels.

Sample	SSA ($m^2.g^{-1}$)	Pore Vol. ($cm^3.g^{-1}$)	Total Porosity (vol.%)	Zeta Potential (mV)	Contact Angle (°)
**AP-Dried gel**	783	2.7	81.2	-	-
**AP-Dried-600**	569	2.6	82.5	-	-
**AP-Dried-800**	400	1.8	81.7	-	-
**AP-Dried-1000**	318	1.4	79.8	-	-
**SC- Dried gel**	689	2.7	75.9	2.7±0.6	149±10.3
**SC- Dried-600**	917	4.9	79.6	-4.6±0.5	127±4.5
**SC- Dried-800**	350	1.6	80.3	-23.5±0.8	40±11.7
**SC-Dried-1000**	274	1.4	80.5	-25.7±0.4	16±6.2

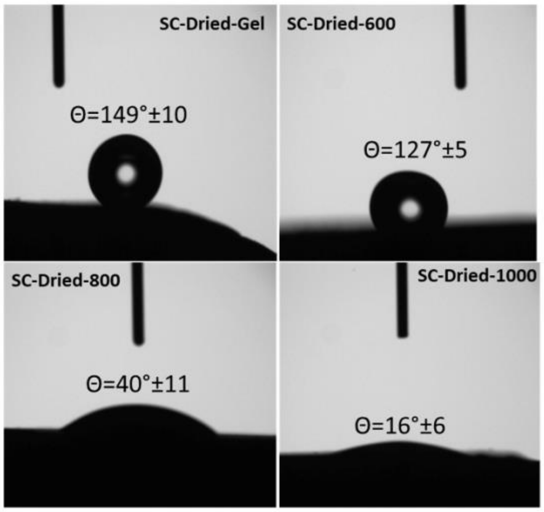

Figure S1. Contact angle images with water droplets on the sample surface.

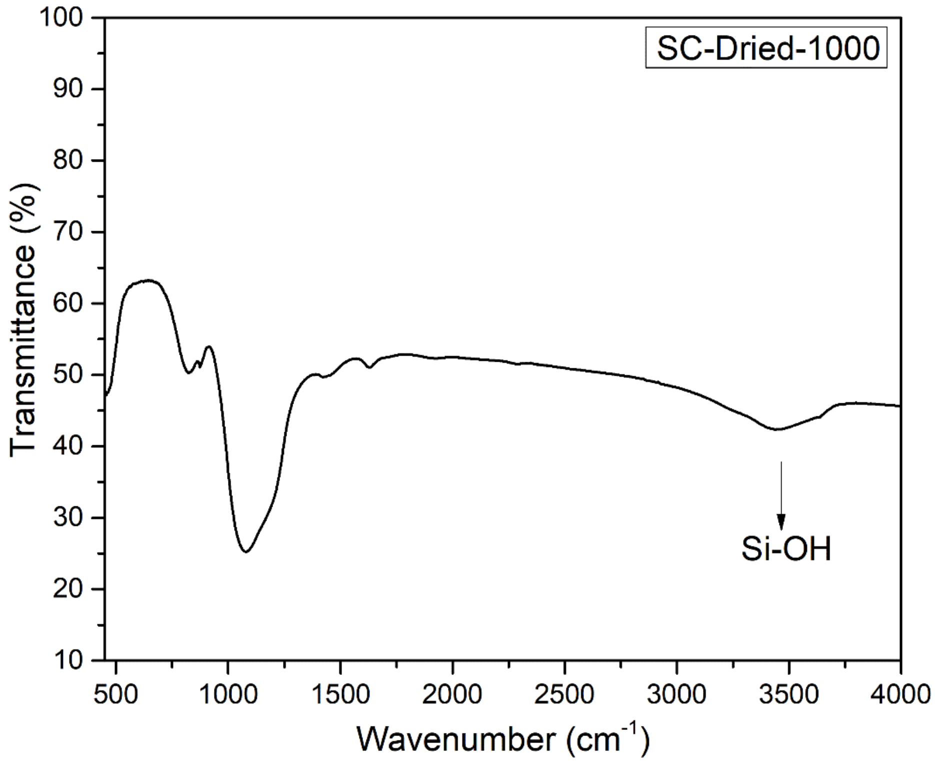

Figure S2. FTIR spectrum of SC-Dried-1000 with no data normalization.

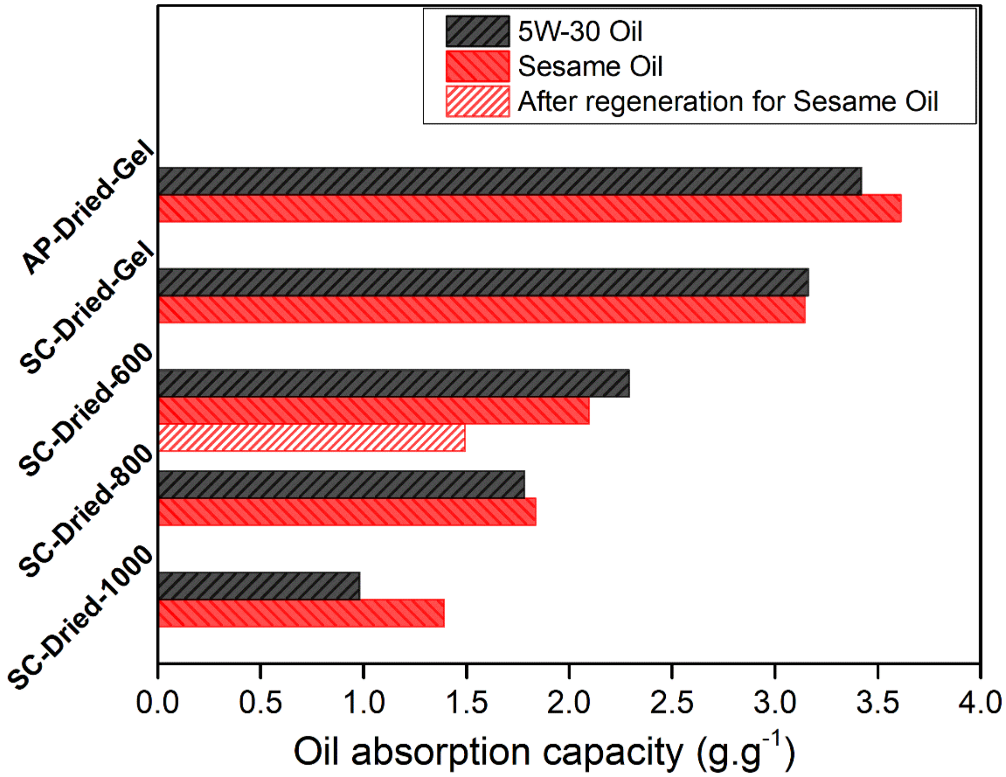

Figure S3. Absorption capacities of ambi/aerogels for sesame and 5W-30 oils.
